# Phase II trial of weekly 24-hour infusion of gemcitabine in patients with advanced gallbladder and biliary tract carcinoma

**DOI:** 10.1186/1471-2407-5-61

**Published:** 2005-06-12

**Authors:** Stefan von Delius, Christian Lersch, Ewert Schulte-Frohlinde, Martina Mayr, Roland M Schmid, Florian Eckel

**Affiliations:** 1 Department of Internal Medicine II, Technical University of Munich, Klinikum rechts der Isar, Ismaninger Str. 22, 81675 München, Germany

## Abstract

**Background:**

Patients with advanced gallbladder and biliary tract carcinoma face a dismal prognosis, as no effective palliative chemotherapy exists. The antitumor effect of gemcitabine is schedule-dependent rather than dose-dependent. We evaluated the activity of a prolonged infusion of gemcitabine in advanced gallbladder and biliary tract carcinomas.

**Methods:**

Nineteen consecutive eligible patients were enrolled. All patients were required to have histologically confirmed diagnosis and measurable disease. Gemcitabine was infused over 24 hours at a dose of 100 mg/m^2 ^on days 1, 8, and 15. Treatment was repeated every 28 days until progression of disease or limiting toxicity. Tumor response was evaluated every second course by computed tomography (CT) scans.

**Results:**

Eighteen patients were evaluable for response. A total of 89 cycles of therapy were administered. One partial response was observed (6%; 95% confidence interval (CI): 0–27%) and ten additional patients had stable disease for at least two months (disease control rate 61%; 95% CI: 36–83%). The therapy was well tolerated, with moderate myelosuppression as the main toxicity. The median time to tumor progression and median overall survival was 3.6 months (95% CI 2.6–4.6 months) and 7.5 months (95% CI 6.5–8.5 months), respectively.

**Conclusion:**

Weekly 24-hour gemcitabine at a dose of 100 mg/m^2 ^is well tolerated. There was a relatively high rate of disease control for a median duration of 5.3 months (range 2.8–18.8 months). However, the objective response rate of this regimen in gallbladder and biliary tract carcinomas was limited.

## Background

Adenocarcinomas of the gallbladder and the biliary tract are relatively uncommon in the western world, with approximately 5,000 cases of gallbladder carcinoma and 2,500 cases of cholangiocarcinoma annually in the USA [1,2]. Worldwide, the highest prevalence of gallbladder cancer is seen in Israel, Mexico, Chile, Japan, and among Native American women, particularly those living in New Mexico. Incidence of cholangiocarcinoma is highest in Israel, Japan, among Native Americans, and in Southeast Asia, where it can reach 87 per 100,000 [3].

Biliary tract tumors can occur anywhere in the hepatobiliary system. Cholangiocarcinoma may be further classified as intrahepatic or extrahepatic (hilar and distal bile duct) carcinomas. Hilar tumors, so called Klatskin's tumors, usually require partial liver resection for cure, whereas distal tumors may require pancreatectomy.

Gallbladder carcinoma has been linked to several risk factors. Gallstones, especially larger than 3 cm, chronic inflammation, bacterial infection, polyps, calcified (porcelain) gallbladder, ulcerative colitis, high energy and total carbohydrate intake, and high body mass index in women increase risk. Risk factors for cholangiocarcinoma are well understood, although most patients lack identifiable risks. Patients with primary sclerosing cholangitis, congenital choledochal cysts (most likely due to associated inflammation and bacterial infection), ulcerative colitis, exposure to carcinogens including cigarette smoke, hepatolithiasis, liver infection, hepatitis C, and biliary parasites carry an elevated risk [3].

Due to the lack of characteristic early symptoms, a definitive diagnosis of gallbladder and biliary tract carcinoma is often established at an advanced stage, and prognosis of patients with advanced tumors remains dismal. Even in patients undergoing aggressive surgery, the general outcome has been disappointing. Five-year survival rates are between 5–10% [1]. Median survival of patients with advanced-stage disease is in the range of only a few months. Due to the lack of randomized phase III studies there is no standard regimen for palliative chemotherapy of gallbladder and biliary tract carcinomas. Depending on the patient's general condition best supportive care, a clinical trial, 5-FU, or gemcitabine is recommended according to guidelines of the National Comprehensive Cancer Network.

Gemcitabine is phosphorylated by deoxycytidine kinase into the active metabolite gemcitabine triphosphate (GemTP). The rate-limiting step in the formation of GemTP is the phosphorylation of gemcitabine to the monophosphate by deoxycytidine kinase [4]. The diphosphate is a potent inhibitor of ribonucleotide reductase, an action that reduces deoxynucleotide pools. Decreased cellular concentrations of deoxycytidine triphosphates permit a more rapid phosphorylation of gemcitabine and decrease the metabolic clearance of gemcitabine nucleotides. As a consequence of this, the active nucleotide forms of gemcitabine are effectively accumulated to high concentrations in the cell [5].

According to the rate-limited activation of gemcitabine a schedule-dependent antitumor effect of gemcitabine was found in various in vivo model systems in preclinical studies [6] suggesting slower infusion rates in the clinical setting. Furthermore, the treatment of mice bearing the colon 26–10 murine colon carcinoma was considerably more effective by applying a weekly 24-hour schedule [7].

Based on the results of a pilot study [8] and a phase II study in patients with pancreatic adenocarcinoma [9] we started a phase II trial of weekly 24-hour infusion of gemcitabine in patients with advanced gallbladder and biliary tract carcinoma.

## Methods

### Patients

To be eligible for the trial, patients were required to have histologically confirmed, irresectable or metastatic carcinoma of the gallbladder or biliary tract. Patients had to have measurable disease on a computed tomography (CT) scan or magnetic resonance imaging (MRI) and had to have a performance status (WHO) ≤ 2. Patients were allowed to have received one previous chemotherapy, but no previous treatment with gemcitabine. They also met the following laboratory criteria: serum bilirubin value ≤ two times upper normal limit, leukocyte count ≥ 3.0 × 10^9^/L, platelet count ≥ 100 × 10^9^/L, and serum creatinine ≤ 2.0 mg/dL.

Patients with active infections, unstable cardiovascular conditions, brain metastases, or other serious medical illnesses were excluded from this trial.

Informed consent was obtained from all patients included in the study, which was approved by local ethics committees.

#### 

### Treatment Plan

Therapy was administered on an outpatient basis and patients were premedicated with appropriate antiemetics.

Patients received gemcitabine (100 mg/m^2^) as a 24-hour intravenous (i.v.) infusion. Treatments were repeated every week for three consecutive weeks followed by one week of rest; a full cycle consisted of four weeks.

Doses were modified according to the following criteria: The weekly gemcitabine dose was reduced by 25% for patients whose leukocyte count was  ≤ 2.5–3.0 × 10^9^/mL, whose platelet count was ≥ 75–100 × 10^9^/L, or if grade 2 nonhematologic toxicity was present. The weekly dose was reduced by 50% for patients whose leukocyte count was ≤ 2.0–2.5 × 10^9^/L or whose platelet platelet count was ≥ 50–75 × 10^9^/L. The weekly dose was omitted if the leukocyte count was less than 2.0 × 10^9^/L, platelet count was less than 50 × 10^9^/L, or grade 3 nonhematologic toxicity was present.

### Patient Evaluation

Weekly blood counts were obtained to determine the level of myelosuppression. Before each cycle a full clinical evaluation noting the performance status and a physical exam were performed and a complete chemistry panel was obtained. In addition, CA19-9 levels were measured every eight weeks.

The antitumor response was evaluated every eight weeks by CT or MRI, unless signs of progression were evident. In terms of response RECIST criteria [10] were applied, since in most cases of these tumors interpretation of bi-dimensional measurements is complicated by associated inflammation and/or necrosis, as well as anatomical structures in the vicinity. Complete response was defined as the disappearance of all signs and symptoms of disease. Partial response was defined as a decrease of > 30 % of the sum of the largest diameters of target (= measurable) lesions without appearance of new lesions or progression of non-target (= evaluable) lesions. To be assigned a status of response, changes in tumor measurement were confirmed by repeat assessment that was performed no less than 4 weeks after the criteria for response were first met. Stable disease was defined as no sufficient shrinkage to qualify for partial response or less than a 20% increase in the sum of the largest diameters of target lesions without appearance of new lesions or progression of non-target lesions. Progressive disease was defined as a 20% increase in the sum of the largest diameters of target lesions or as appearance of new lesions or as progression of non-target lesions.

The response was validated by radiologists independent of the study.

Disease control was defined as the absence of tumor progression (i.e. complete and partial response and stable disease) for at least two months.

### Statistical Methods

The primary end point of this trial was objective response. Secondary end points included progression free survival, overall survival, and toxicity.

Simon's optimal two-stage design was used for calculation of the sample size [11]. Early stopping rules were provided to allow for study discontinuation in the event of lack of efficacy. Sufficient responses (greater than 2 in 18 evaluable patients) were required to trigger the second phase of enrollment for another 17 for a maximum of 35 patients. 95% confidence interval was calculated by the method of Clopper and Pearson. Correlations are nonparametric by the method of Spearman. Progression free survival and overall survival were calculated from the start of chemotherapy until progression, death or last follow-up by the Kaplan-Meier method using SPSS, version 12.0 software.

## Results

A total of 19 patients – eight men and eleven women – were entered onto this trial. Their pre-treatment characteristics are listed in Table 1. The median age was 63 years (range 30–83 years, 14 patients younger than 75 years). All patients had adenocarcinomas. There were three patients with gallbladder carcinoma, all of whom were women. Four patients (21%) had relapse of disease after surgery with incomplete resections in two patients. Two patients (11%) had been previously treated with a combination of cyclophosphamide, leucovorin, 24-hour fluorouracil and tamoxifen [12]. All other patients were chemotherapy-naïve. Seven patients (37%) required biliary tract decompression by endoscopic or percutaneous stenting because of obstructive jaundice before the start of chemotherapy. Baseline CA19-9 levels were recorded in 17 patients and were elevated in 14 of them. One female patient was not evaluable regarding response due to early discontinuation of therapy because of withdrawal of consent.

### Toxicity

A total of 89 cycles were administered during the trial (median 4 cycles; range 1–19 cycles). Overall, weekly 24-hour gemcitabine was well tolerated (Table 2). No treatment-related deaths occurred. Myelosuppression was the major toxicity. Among all 19 patients grade 3/4 neutropenia occurred in four patients (21%) with no episode of febrile neutropenia. One patient developed grade 4 thrombocytopenia with epistaxis and needed a platelet transfusion. No grade 4 and only one case of grade 3 nonhematologic toxicity effects were noted. Nausea and emesis, when present, usually were mild. Grade 3 mucositis was present in one patient. Allergic urticaria following gemcitabine infusion was seen in one patient, but was manageable by antiallergic medication during the further course.

### Response and Survival

No complete response was observed. One of the 18 evaluable patients (6%; 95%CI: 0–27%) with a gallbladder carcinoma had a partial response. Ten additional patients had stable disease for a median duration of 5.3 months (range 2.8–18.8 months) resulting in a disease control rate of 61% (95%CI: 36–83%). Two of these patients had gallbladder carcinoma, four intrahepatic cholangiocarcinoma, and four extrahepatic cholangiocarcinoma. Seven (39%, 95%CI: 17–64%) patients had stable disease after 4 cycles of chemotherapy.

**Figure 1:**
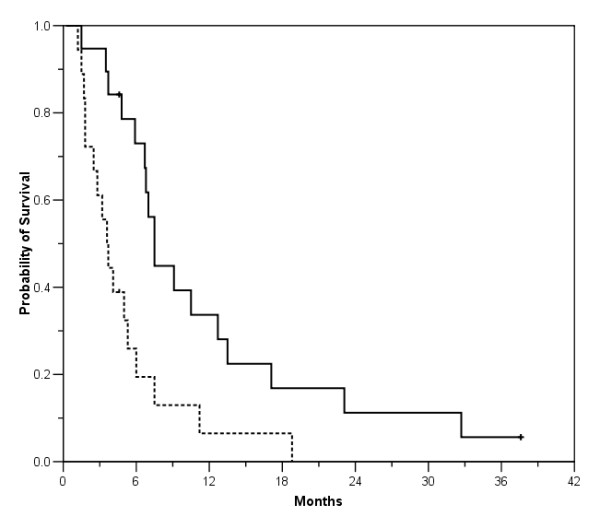
Probability of progression free survival (dotted line) and overall survival for all 19 patients enrolled.

Median progression free survival was 3.6 months (95% CI 2.6–4.6 months) with a 3 and 6-month progression free survival rate of 61% and 23%, respectively. Median overall survival for all 19 patients was 7.5 months (95% CI: 6.5–8.5 months) with a 6 and 12-month survival rate of 73% and 34%, respectively (Figure 1). There was a significant correlation between progression free and overall survival (r = 0.54, p = 0.016).

In fourteen of seventeen patients (82%) assessed baseline CA19-9 levels were elevated (median 581, mean 98,849, range 115–1,244,000; normal < 32 U/mL). In eleven patients follow-up values were obtained. Ten of these eleven patients had elevated baseline levels. There was a significant negative correlation (r = -0.65, p = 0.032) between relative changes of CA19-9 during chemotherapy compared with baseline and progression free survival.

## Discussion

In this phase II trial of a prolonged infusion of gemcitabine for advanced gallbladder and biliary tract carcinoma we only found a 6% objective response rate (95%CI: 0–27%). According to the early stopping rules accrual was terminated after enrollment of 19 patients, thereof 18 evaluable for response. Disease control for at least two months was observed in 11 patients (61%, 95%CI: 36–83%) for a median duration of 5.3 months (range 2.8–18.8 months). As expected there was a significant correlation of progression free and overall survival. Toxicity was generally mild with myelosuppression as the major toxicity.

Only relatively small phase II trials have assessed the efficacy and toxicity profiles of chemotherapy regimens in the palliative treatment of gallbladder cancer and cholangiocarcinoma. Due to tumor-specific complications such as obstructive jaundice with impaired hepatic metabolism and biliary excretion toxicity profiles of chemotherapy regimens may be different in gallbladder and biliary tract carcinoma compared to other cancers. Single-agent and multiagent regimens have yielded modest results in patients with advanced biliary carcinomas. Overall response rates and disease control rates are about 25% (mostly 13–35%, range 0–64%) and about 65% (mostly 55–72%, range 19–94%), respectively [13].

Among several different new anticancer drugs, gemcitabine has generated particular interest. Considered as standard treatment for advanced pancreatic carcinoma [14] and given the histogenetic affinity between the pancreas and the biliary tract, a number of phase II trials of gemcitabine with differing results were conducted in patients with advanced gallbladder and biliary tract cancers. Patients mostly received gemcitabine at a dose of 1000 mg/m^2 ^over 30 minutes.

Possible options to further improve the therapeutic efficacy of gemcitabine monotherapy include modifications of the administration schedule. Tempero et al. [15] found improved activity of gemcitabine 1500 mg/m^2 ^as a fixed dose rate infusion of 10 mg/m^2^/min in comparison to high dose gemcitabine (2200 mg/m^2^) infusion over 30 minutes in pancreatic cancer. Pharmacokinetic analysis in this study also revealed significantly higher concentrations of intracellular gemcitabine triphosphate with the prolonged infusion. However, grade 3–4 hematologic toxicity occurred in up to almost 50% in the fixed dose rate arm. In contrast, in our trial of 24-hour gemcitabine at a dose of 100 mg/m^2 ^toxicity was mild.

Median survival achieved by our patients was 7,5 months (95% CI: 6.5–8.5) and therefore in the range of most other studies investigating gemcitabine as single agent for biliary tract cancer. Progression free survival (3.6 months; 95% CI 2.6–4.6) was similar to other published data of phase II trials of gemcitabine in biliary tract cancer [Table 3]. Nevertheless, with only small objective response observed our results do not support the use of gemcitabine as continuous infusion in this setting.

## Conclusion

In conclusion, weekly 24-hour infusion of gemcitabine at a dose of 100 mg/m^2 ^in patients with advanced gallbladder and biliary tract carcinoma can be safely administered in an outpatient setting. A relatively high disease control rate was observed in this trial. However, in terms of objective response antitumor activity was only marginal. Therefore, gemcitabine as continuous infusion seems not to be superior to gemcitabine administered in short term.

## Competing interests

The author(s) declare that they have no competing interests.

## Authors' contributions

SvD carried out the care of the patients, collection and interpretation of the data, and drafting of the manuscript. CL conceived of the study, and participated in its design and coordination. ES-F participated in its design and coordination. MM participated in patient care and coordination. RMS helped to draft the manuscript and to interpret the results. FE conceived of the study, participated in its design and coordination and carried out the statistical analysis. All authors read and approved the final manuscript.

## Pre-publication history

The pre-publication history for this paper can be accessed here:



## Figures and Tables

**Table 1:** Patient characteristics of all patients enrolled.

Total no. of patients (evaluable for response)	19 (18)
Sex (female/male)	11/8
Age, median (range)	63 (30–83)
Performance status (WHO)	
0	1 (5%)
1	12 (63%)
2	6 (32%)
Tumor site	
Gallbladder	3 (16%)
Intrahepatic bile ducts	9 (47%)
Extrahepatic bile ducts	7 (37%)
Site of metastasis	
Liver	9 (47%)
Lung	2 (11%)
Lymph nodes	5 (26%)
Peritoneum	2 (11%)
Recurrence after previous surgical resection	4 (21%)
Pre-treatment requiring stent or percutaneous transhepatic drainage because of obstructive jaundice	7 (37%)
Pre-treatment chemistries	
Total bilirubin (mg/dl)	
Median	0,7
Range	0,3–2,3
Gamma-glutamyltransferase (<66 U/L^a^)	
Median	159
Range	34–671
Aspartate aminotransferase (10–50 U/l^a^)	
Median	29
Range	8–145
CA19-9 (<32 U/mL^a^)	
Median	387
Range	4-1244000

^a ^normal levels

**Table 2:** Treatment-related toxicities of all 19 patients enrolled.

	Number of patients (%)			
NCI-CTC Grade	1	2	3	4
Leukopenia	2 (11)	6 (32)	3 (16)	0
Neutropenia	3 (16)	1 (5)	2 (11)	2 (11)
Anemia	0	1 (5)	0	0
Thrombocytopenia	3 (16)	3 (16)	0	1 (5)
Diarrhea	1 (5)	1 (5)	0	0
Nausea/emesis	5 (26)	2 (11)	0	0
Stomatitis	3 (16)	2 (11)	1 (5)	0

NCI-CTC: National Cancer Institute Common Toxicity Criteria

**Table 3:** Selection of phase II studies of gemcitabine in advanced biliary tract cancer.

Study	Number of assessable patients	Response	SD	PFS (months)	OS (months)
Metzger et al (16)	13	1 (8%)	11 (85%)	7	16
Verderame et al (17)	4	1 (25%)	3 (75%)	11	NR
Gebbia et al (18)	18	4 (22%)	5 (28%)	3	8
Penz et al (19)	32	7 (22%)	14 (44%)	6	12
Lin et al (20)	24	3 (13%)	8 (33%)	3	7
Eng (21)	14	0 (0%)	2 (13%)	2	5
Tsavaris et al (22)	30	9 (30%)	11 (37%)	7	14
Park et al (23)	23	6 (26%)	8 (35%)	8	13

Abbreviations: NR, not reported; OS, overall survival; PFS, progression-free survival; SD, stable disease.
